# Ultra-Sensitive Isopropanol Biochemical Gas Sensor (Bio-Sniffer) for Monitoring of Human Volatiles

**DOI:** 10.3390/s20236827

**Published:** 2020-11-29

**Authors:** Po-Jen Chien, Takuma Suzuki, Ming Ye, Koji Toma, Takahiro Arakawa, Yasuhiko Iwasaki, Kohji Mitsubayashi

**Affiliations:** 1 Institute of Chemistry, Academia Sinica, No. 128, Sec. 2, Academia Rd., Nankang, Taipei 115, Taiwan; wizgx@gate.sinica.edu.tw; 2 Graduate School of Medical and Dental Sciences, Tokyo Medical and Dental University, 1-5-45 Yushima, Bunkyo-Ku, Tokyo 113-8549, Japan; sensinglabo@gmail.com; 3 Department of Biomedical Devices and Instrumentation, Institute of Biomaterials and Bioengineering, Tokyo Medical and Dental University, 2-3-10 Kanda-Surugadai, Chiyoda-ku, Tokyo 101-0062, Japan; ym@fujiclinic.jp (M.Y.); toma.bdi@tmd.ac.jp (K.T.); arakawa.bdi@tmd.ac.jp (T.A.); 4 Faculty of Chemistry, Materials and Bioengineering, Kansai University, 3-3-35 Yamate-Cho, Suita-Shi, Osaka 564-0836, Japan; yasu.bmt@kansai-u.ac.jp

**Keywords:** breath isopropanol, biosensor, NADH, secondary alcohol dehydrogenase, volatile organic compounds, gas sensor

## Abstract

Our groups have previously developed a biochemical gas sensor to measure isopropanol (IPA) in exhaled air and have applied it for breath IPA investigation in healthy subjects and diabetes patients. In this study, the original bio-sniffer was modified with a series of components that improved the limit of detection (LOD). First, the modified IPA bio-sniffer used a C8855-type photomultiplier tube (PMT) that performed well in the photon sensitivity at the peak wavelength of nicotinamide adenine dinucleotide (NADH) fluorescence. Second, the multi-core bifurcated optical fiber, which incorporated 36 fibers to replace the previous dual-core type, enhanced the fluorescence collection. Third, the optical fiber probe was reinforced for greater width, and the flow-cell was redesigned to increase the area of the enzyme-immobilized membrane in contact with the air sample. These modifications lowered the detection limit to 0.5 ppb, a significant increase over the previous 1.0 ppb. Moreover, the modified bio-sniffer successfully analyzed the IPA concentration in exhaled air from a volunteer, which confirmed its capability for real-world sample detection. The modified bio-sniffer is more applicable to breath measurement and the detection of other extremely-low-concentration samples.

## 1. Introduction

Human gas—breath, body odor, mouth odor, urine vapor, and skin—has attracted considerable attention in the development of non-invasive diagnosis systems because of its numerous known biomarkers and potential disease cues [1,2,3]. The analysis of biological gases enables non-invasive diagnostic, beneficial for preventative medicine, and metabolic assessment [4,5]. Exhaled air is one of the central analysis targets because it is much easier to collect than other samples. Breath volatile organic compounds (VOC) are produced by blood and alveoli [6,7], providing more direct information about body conditions. Some biomarkers in the breath have been applied to clinical use. For example, the concentration of exhaled nitric oxide can be used to diagnose and monitor the treatment state of chronic asthma [8,9]. Furthermore, many other VOCs in the breath are under study as potential biomarkers. For instance, the acetone concentration in the breath received extensive investigation of its correlation with blood glucose concentration [10,11,12,13] and could serve as an indicator of ketosis in adult ketogenic diets [14]. Moreover, the alteration of the breath isoprene concentration has been reported to be related to lung cancer [15,16]. Therefore, creating a breath analysis system is vital to medicine and healthcare.

Breath analysis methods include gas chromatography-mass spectrometry [17], real-time mass spectrometry [18], gas chromatography-tandem mass spectrometry [19]. The mass spectrometry methods are usually promising because of their highly sensitive, selective, multi-target analysis capabilities. However, their expensive cost, time requirements, and requisition of professional operation form barriers in general clinical use and research. Therefore, many semiconductor-based breath analysis sensors, electronic noses, and algorithms flourish [20,21,22,23,24]. The enzyme-based biosensing techniques provide another choice for breath analysis. In our previous work, we developed a highly sensitive biochemical gas sensor with high selectivity, called bio-sniffer, to measure gaseous isopropanol (IPA) in human breath [25]. IPA is an acetone-related metabolite and may also be related to ketoacidosis, diabetes, and breast cancer [26,27,28]. Our IPA bio-sniffer used the fluorescence emitted from the reduced form of nicotinamide adenine dinucleotide (NADH), an enzymatic reaction product of the secondary alcohol dehydrogenase (S-ADH) as the signal. In a weak alkaline condition, approximately pH 8.5 to pH 9, S-ADH tends to catalyze IPA to acetone and reduce NAD^+^ (oxidized form of nicotinamide adenine dinucleotide) to NADH. NADH has a specific optical property: it releases fluorescent light with a peak wavelength at 490 nm when excited by 340 nm ultraviolet. Accordingly, the increase of the NADH fluorescence intensity can be useful to measure the IPA concentration. Previously, the developed IPA bio-sniffer was applied together with our acetone bio-sniffer to investigate the exhaled IPA and acetone in healthy subjects and diabetes mellitus patients. We found that both breath IPA and acetone exhibited higher concentrations in diabetes mellitus patients [29]. However, during the breath IPA measurement experiment, we noticed that some samples from healthy volunteers had IPA concentrations lower than the detection limit [25]. This result indicated that the IPA concentration in healthy people might be extremely low, at less than 1 ppb, consistent with Claire Turner’s report [30]. Thus, improving the detection limit is necessary for expanding the bio-sniffer application fields.

In this study, the previously developed bio-sniffer was modified, aiming at a detection limit lower than 1 ppb. The IPA bio-sniffer comprises three components: a gas source transport, an NADH fluorescence detection system, and a flow-cell with an S-ADH immobilized membrane. The performance of all components was considered to be improved for achieving the goal. Because the bio-sniffer used NADH fluorescence as the signal, we first tried to increase the detection ability of the NADH fluorescence intensity. The NADH fluorescence detection instrument contained three components: (1) a photon detection unit (photomultiplier tube, PMT), used to measure the fluorescent intensity, (2) an ultraviolet light-emitting diode(UV-LED), used as the excitation light source, and (3) a bifurcated optical fiber to transmit the excitation light and collected fluorescence. Thus, we compared the different PMT models and produced a customized bifurcated fiber. Second, we considered increasing the bio-sniffer sensing region because contact with more IPA molecules simultaneously could cause more NADH to be produced. Therefore, the optical fiber probe was strengthened to enhance fluorescence collection, and the flow-cell was redesigned. The performance of the modified IPA bio-sniffer improved significantly with these modifications.

## 2. Materials and Methods

### 2.1. Materials and Reagents

Hydrochloric acid, 2-amino-2-hydroxymethyl-1,3-propanediol (Tris, 99.9%, Biochemistry grade) and isopropanol (99.7%, JIS special grade) solution was purchased from Wako Pure Chemical Industries, Japan. The ß-NAD^+^ powder was bought from Oriental Yeast, Co., Ltd., Tokyo, Japan. NADH-dependent secondary alcohol dehydrogenase (S-ADH, EC 1.1.1.x, 1 unit/mg) from yeast was obtained from Daicel Chiral Technologies, Co., Japan. Hydrophilic polytetrafluoroethene filter membrane (H-PTFE, pore size: 0.2 µm, porosity: 80%, JGWP14225) employed for S-ADH immobilization was acquired from Millipore, USA. The PMEH polymer (poly (MPC-co-2-ethylhexyl methacrylate)) was synthesized from 2-methacryloyloxyethyl phosphorylcholine co-polymerized with 2-ethylhexyl methacrylate, and the particulars information of the preparation PMEH polymer was described in our previous study [31].

### 2.2. Fabrication and Principle of IPA Bio-Sniffer

**Figure 1:**
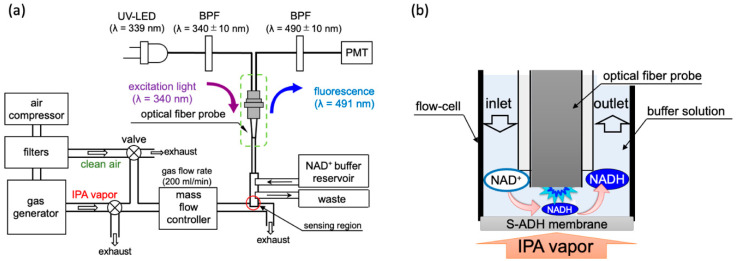
(**a**) Sketch of the IPA bio-sniffer system. It contains a gas generator system, NADH detection system, and the flow-cell with an enzyme membrane. The flow rate of the gas, including standard IPA vapor and the breath sample, was set at 200 mL/min, adjusted by a control valve and a mass flow controller. (**b**) Sensing principle of the IPA bio-sniffer.

The details of the IPA bio-sniffer information and characterizations—such as S-ADH selectivity, reproducibility, sensor optimization, and the enzyme-immobilized membrane property—were described in our previous work [25]. As depicted in Figure 1 and described in the Introduction, the IPA bio-sniffer used a UV-LED (Sensor Electronic Technology, Inc., Atlas, CU, USA) connected with the bifurcated optical fibers as an excitation light source. The other end of the bifurcated probe was linked with a PMT to measure the NADH fluorescence intensity. The bifurcated fibers’ joint end was extended with an optical fiber probe to transmit the excitation light and receive the fluorescence signal. Bandpass filters (BPF, λ = 340 ± 10 nm, and 490 ± 10 nm, Asahi Spectra Co., Ltd., Tokyo, Japan) were added to reduce the noise.

The gas sensing region was constructed using a flow-cell and an enzyme stabilized membrane at the optical fiber probe’s tip. The enzyme-immobilized membrane was produced by mixing the S-ADH powder into a PMEH solution and spreading it carefully on an H-PTFE membrane. The prepared membrane would be stored in a 4 °C refrigerator for three hours for enzyme immobilization. Further, 1 cm^2^ of the membrane requires 10 µL of PMEH (10%, w/w in ethanol) and 1.25 unit of S-ADH. The gaseous IPA diluted in air at known concentrations was produced using two types of gas generators. One was an off the shelf model (PD-1B-2, Gastec, Co., Ltd., Kanakawa, Japan) that can produce an IPA concentration from 7.5 ppb to 54 ppm. The other was a custom-made machine created by Gastec, Co., Ltd., Kanakawa, Japan, which could generate a gaseous IPA concentration from 0.5 to 10 ppb. The principle of generating different concentrations of IPA gas is described in Supplementary Materials. Because the IPA gas concentration was controlled by diluting the IPA vapor in carrier air, the carrier gas interference should be eliminated. The carrier gas was generated by an air compressor collecting gas from the environment and flowed through three filters, including an activated carbon filter, a ketone-and-alcohol absorption filter, and a dehumidification filter. All gases were delivered via Teflon tubes. In front of the sniffer sensing region, a mass flow controller (RK1200, Koflok Inc., Kyoto, Japan) was used to adjust the airflow rate, set to 200 mL/min. Two switch valves were placed so as to close either the way out of the gas generator or the clean air output in order to select the gas going through the sensing region. During the measurement period, a Tris-HCl buffer with 100 mM NAD^+^ was continuously supplied to the flow-cell at a flow rate of 1.5 mL/min by a liquid chromatography pump (SP-21–32, FLOM Co. Ltd., Tokyo, Japan) to continuously moisten the S-ADH membrane, provide reaction materials, and eliminate the produced products. The sensing principle is shown in Figure 1b. When diluted IPA vapor flowed through the sensing region, the S-ADH catalyzed NADH production, and the PMT instantly detected the change in fluorescence. The buffer flow regularly cleaned out the NADH and introduced new NAD^+^. Therefore, the bio-sniffer measured the IPA concentration based on the generation and eradication velocity of NADH.

### 2.3. Description of Two Types of PMT and the Multi-Core Bifurcated Optical Fiber

The first of the sensitivity improvements we tested compared two different PMT models: C9692 and C8855 (C8855 is a counting unit combined with H7421-40 photon counting head. In addition, temperature controllable power supply is C8137). Both were bought from Hamamatsu Corporation, Japan. C9692 was the PMT applied in our previous acetone bio-sniffer [32] but never used in the IPA bio-sniffer. The two PMTs were installed on the same NADH fluorescence detection system, as depicted in Figure S1, and measured the fluorescence of standard NADH at a concentration of 1 µM. Before the test, the optical probe was stepped into a dark cuvette that contained 300 µl of blank Tris-HCl buffer solution and waited until the background noise intensity stabilized. We then dropped 3 µl of NADH stock solution into the cuvette, for a final concentration of 1 µM. The fluorescence detection period was set to 5 min, and the signal was defined as the average intensity measured from 4 min 20 s to 4 min 50 s after NADH introduction.

**Figure 2:**
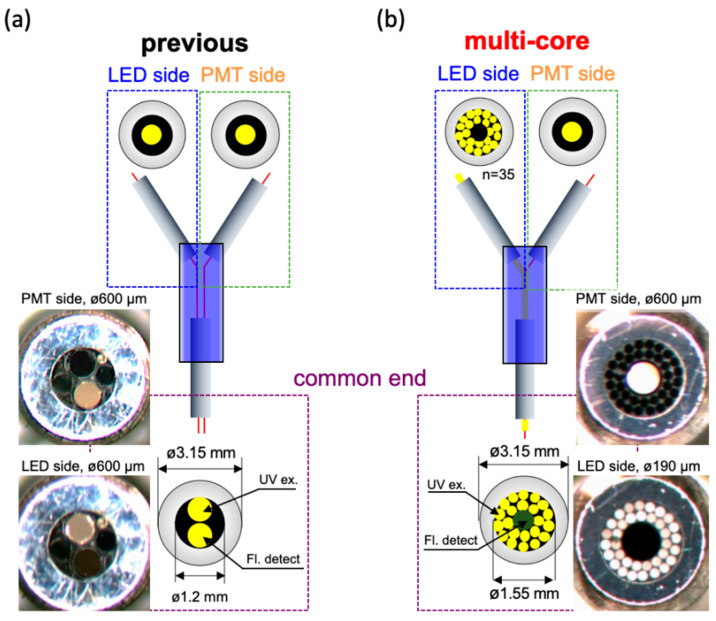
Schematic diagram and pictures of bifurcated optical fiber probe. (**a**) The dual-core type of fiber. The diameters of LED and PMT connected fibers were both 600 µm; (**b**) The multi-core type of fiber. The diameter of PMT connected fiber was still 600 µm and surrounded by 35 LED light transmitted fibers with each diameter was 190 µm.

The second component that was change was the bifurcated fiber. The original bifurcated fiber was a dual-core type (BIF600-UV/VIS) obtained from Ocean Optics Inc. Orlando, FL, USA. As described in Figure 2a, the dual-core type possesses only two fibers: one connected to the LED and the other linked to the PMT. The diameters of both fibers were 600 µm, and they were packaged into the common end with a 1.2 mm diameter. For enhancing the efficiency of excitation light transmission, we adopted the multi-core type bifurcated fiber, which was custom-made from Mitsubishi Cable Industries, Ltd. Japan. In this layout, the diameters of the fiber that connects to the PMT remain at 600 µm. However, the excitation light connected end was modified from one thread to 35 fibers, in which the diameters were 190 µm for each one. As depicted in Figure 2b, the common end is bundled up by 36 fibers, in which the LED-transmission fibers surround the fluorescence receiver fiber in the center. Consequently, the diameter of the common end increased to 1.55 mm. For evaluating their performance, the two bifurcated fibers were applied to measure a series of NADH solutions with known concentrations.

### 2.4. Enlarged Fiber Probe and Redesigned the Flow-Cell

**Figure 3:**
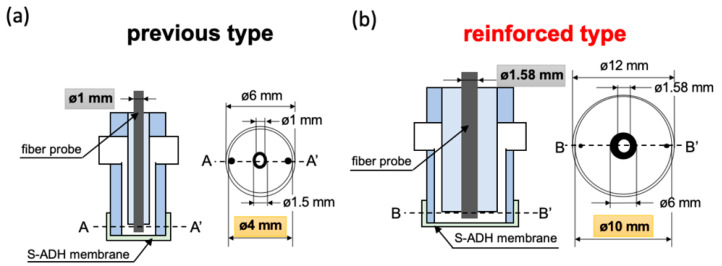
Schematic diagram of the bio-sniffer sensing region. (**a**) The previous design. The diameter of the fiber probe was 1 mm, and of the flow-cell was 4 mm. (**b**) The enlarged design. The diameter of the fiber probe was 1.58 mm, and of the flow-cell was 10 mm.

The third component for modification was widening the diameter of optical fiber probe, which was used to extend the fluorescence collection ability. Additionally, we also redesigned the flow-cell to function in concert with the fiber probe. This modification increased the bio-sniffer’s sensing region. Figure 3a illustrates the previous structure of the flow-cell. The diameter of the optical fiber probe was 1 mm (F1000-ANGLE90, Ocean Optics Inc. Orlando, FL, USA), which could not entirely cover the fiber area (Figure 2b, 1.2 mm). The flow-cell was produced from a silicone tube, and a polymethylmethacrylate (PMMA) tube with a final diameter of 4 mm in contact with the enzyme membrane. In this study, the reinforced enlarged fiber was custom-made by Mitsubishi Cable Industries, Ltd., Japan. The diameter was increased to 1.58 mm for matching the fiber area at the common end of the multi-core type fiber. The redesigned flow-cell, which was produced in PMMA, is depicted in Figure 3b. The area in contact with the S-ADH enzyme membrane is significantly increased, with a diameter of 10 mm.

### 2.5. Procedure for the Bio Sniffer Calibration Range Measurement

The modified IPA bio-sniffer was used to detect diluted IPA at known concentrations so as to evaluate the bio sensor detection range. Before measuring IPA concentration, the carrier air as a blank was flushed into the Teflon tube until the background noise stabilized. The valves were then switched to enable the diluted IPA to be delivered to the sensing region for 10 min to measure the signal. Finally, the valves were switched again to flow the pure air through for washing out the residue IPA vapor in the tubes. The modified IPA bio-sniffer measured the standard diluted IPA vapor with concentration from 0.5 to 9060 ppb so as to determine the detection range and compare it with the previous biosensor performances. The limit of detection (LOD) was defined as a signal that is at least three times higher than the standard deviation of the blank test measurement, which was the noise detected from the carrier air.

### 2.6. Procedure for the Breath Sample Measurement

For evaluating the ability of modified IPA bio-sniffer in a real-world sample test, a human breath sample collected from a volunteer was analyzed by the sniffer. The volunteer was required to comply with the following requirements: (1) no smoking within an hour before the breath collection, (2) no drinking of alcoholic beverages or use of any oral spray containing alcohol within 24 h, and (3) no starvation or strenuous exercise within 24 h. The procedure to gather exhaled air was referenced from Bikov’s report [33]. The subject took a deep inhalation first and held the breath for at least 15 s. Then, the subject was asked to exhale the breath smoothly through a Teflon tube with a three-way valve—the tube could route the breath flow to the sample bag or an exhaust hole. The breath during the first three seconds was not to be collected and be discarded through the exhaust port. We then switched the valve to allow the remaining breath to enter a two-liter gas collection bag (2–9981–03, As one Co., ltd., Osaka, Japan). The gathered sample was immediately analyzed by the modified IPA bio-sniffer. The bio-sniffer would calibrate before and after the breath experiment. The breath sample measurement experiment was authorized by the Human Investigations Committee of the Institute of Biomaterials and Bioengineering, Tokyo Medical and Dental University (authorization code: 2015−06) in accordance with the Declaration of Helsinki.

## 3. Results and Discussion

### 3.1. The Improvement of NADH Measurement

**Figure 4:**
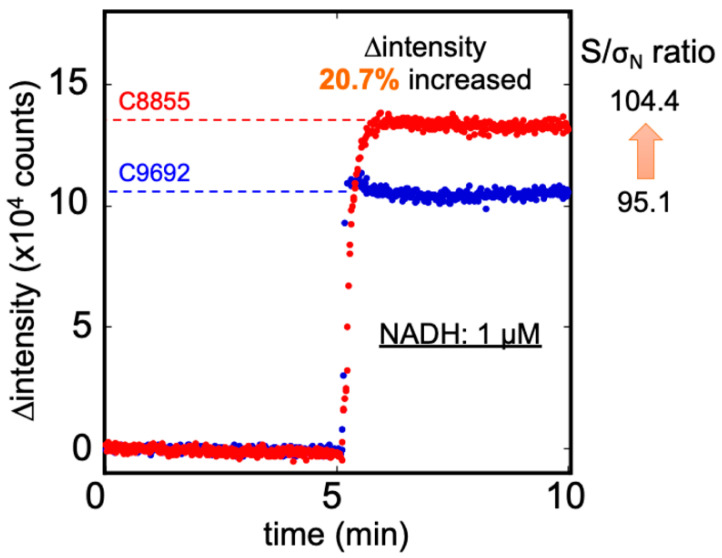
The ∆ intensity of 1 µM NADH was measured by C8855 (red) and C9692 (blue) PMT. The C8855 PMT presented a more 20.7% signal than the C9692 type, and the S/σ_N_ ratio also increased from 95.1 to 104.4.

Figure 4 illustrates the fluorescence intensity of 1 µM NADH detected by C9692 and C8855 PMT. The signal was defined as ∆intensity (the difference between the measured fluorescence intensity and blank buffer), and the unit was counts. The signal intensity measured by C8855 was approximately 12,987 counts, presented a signal 21% higher than with the C9692 PMT (9997 counts). The standard deviations of blank were similar in both PMTs: C9692 was 105 and C8855 was 124. Therefore, the signal-to-noise (S/σ_N_ ratio at 1 µM NADH of C8855 was 104.4, and C9692 was 95.1. The improvement of S/σ_N_ was about 10%. Accordingly, the C8855-type PMT was retained in the IPA bio-sniffer. Table S1 presents the specifications of C8855, H7421-40, and C9692. For the bio-sniffer, the most crucial characteristic of PMT was the count sensitivity at 491 nm wavelength. This result indicated that C8855 with H7421-40 performed better count sensitivity at 491 nm.

The NADH calibration curves were determined by both the dual-core bifurcated fiber with the original fiber probe (diameter 1 mm) and multi-core type with an enlarged probe (diameter 1.58 mm). As depicted in Figure S2, the LOD and calibration range in the NADH measurement did not improve using the multi-core type bifurcated fiber. However, the fluorescence intensities detected in those NADH concentrations over 10 nM were 110% higher, on average, than the original dual-core type fiber. The higher fluorescence intensity at the same NADH concentration was caused by a higher excitation light intensity transferred by the multicore fiber because the area connected with the UV-LED was approximately 3.6 times that of the dual-core type. The power of excitation light detected at the common end of the dual-core fiber was 27.1 µW. On the other side, the multi-core type transfer was approximately 38.7 µW, or 40% higher than the original. It represented that the power of transferred excitation light could be elevated by expanding the connected fiber area. Another factor was that the original fiber diameter (1.0 mm) could not cover the dual-core type area (diameter 1.2 mm). That means both excitation light and fluorescence had a loss at the common end and fiber probe joint. The enlarged fiber probe (diameter 1.58 mm) could entirely cover the multi-core bifurcated fiber. It reduced the loss of excitation light and the collected fluorescence. Considering the common end diameter, we selected a bundle of 35 small fibers instead of a bigger one. If using a bigger fiber, the joint end’s diameter will increase to at least 1.725 mm. That means a more massive fiber probe and larger flow-cell are required.

### 3.2. The Improvement of Diluted IPA Gas Measurement

**Figure 5:**
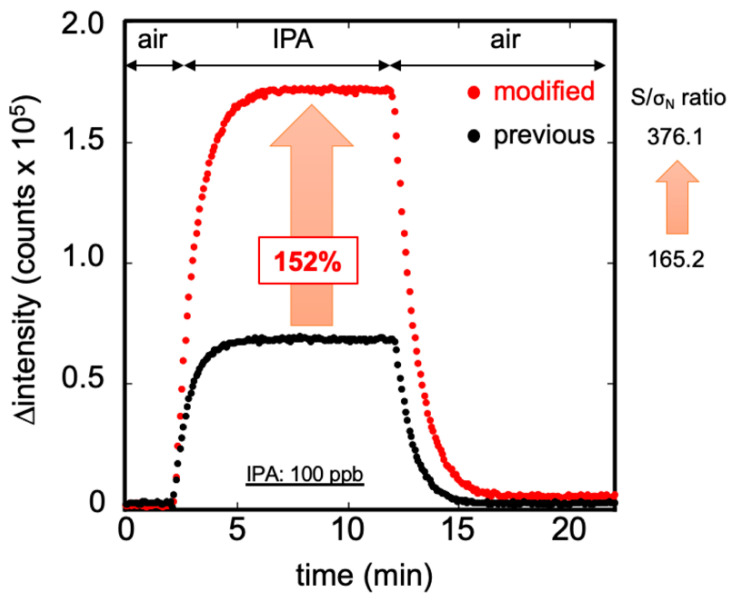
Measurement of 100 ppb standard IPA gas by the previous (black) and the modified (red) bio-sniffer. The modified bio-sniffer showed the signal is higher than with the previous one by 152%, and the S/σ_N_ ratio increased from 165.2 to 376.1. The broader area of the flow cell leads to detection of more NADH for the same IPA concentration but also results in a longer time response and recovery time.

Figure 5 displays the results obtained when measuring IPA gas at 100 ppb, using the previous and modified bio-sniffer. The first 2 min represent the stable condition of the background noise measured from the carrier air. The next 10 min represent the signal detected from 100 ppb IPA vapor. The final 10 min show that the recovers to the initial state when using the carrier air to flush out the residue IPA vapor. At the same IPA concentration measurement, the redesigned bio-sniffer detected a higher signal than the previous one. The fluorescence measured by the reinforced redesigned was approximately 152% higher than the previous one. Moreover, the S/σ_N_ ratio increased from 165.2 to 376.1. Because the bio-sniffer used the dynamic balance between the generation and removal velocity of NADH to estimate the IPA concentration, it requires some time to achieve steady-state. Here, we defined the response time: the time that fluorescence intensity reaches 90% of plateau value. The recovery time was defined as when fluorescence intensity decreases to 10% higher than the blank value. The response time of the original bio-sniffer was 150 s, and the recovery time was 200 s. On the other hand, the modified bio-sniffer required 180 s of response time and 230 s recovery time.

There are two reasons for the dramatic increase of fluorescence signal detected by the modified bio-sniffer. The first is the improved performance of the NADH measurement mentioned above. The second is that the new flow-cell has a more extended area than the previous one and the fiber probe is larger by 58%, enabling an easier NADH fluorescence collection. The broader space at the flow-cell and enzyme membrane increased the circulated buffer volume, which resulted in a lower exchange efficiency between reacted and fresh buffer solution. Thus, for a same IPA concentration, more NADH will be produced in the flow cell. This characteristic contributes to the high sensitivity of IPA detection. Nevertheless, it delays the bio-sniffer’s reaction rate, requiring more time to achieve steady-state during detection and recovery.

### 3.3. Calibration Range of the Modified IPA Bio-Sniffer

**Figure 6:**
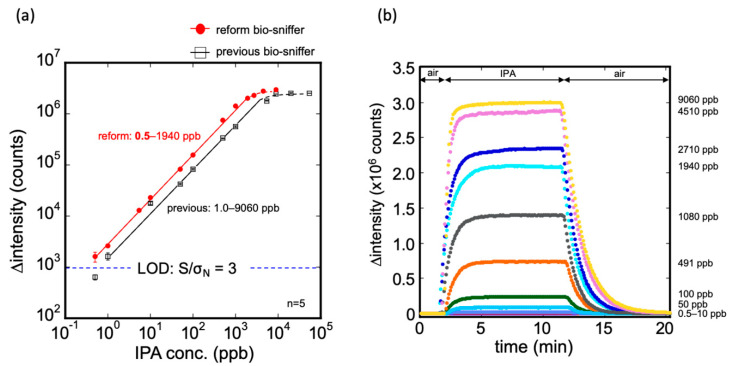
(**a**) Calibration curve of the modified IPA bio-sniffer and comparison with the previous one. The dynamic range of the modified bio-sniffer goes from 0.5 up to 1940 ppb. With the previous sniffer, it was 1.0 to 9060 ppb. The detection limit of the modified bio-sniffer was 0.5 ppb, which is better than the previous 1 ppb; (**b**) Typical responses of the modified bio sniffer to various concentrations of IPA gas.

The calibration curve and the typical response to IPA gas measurement by the modified bio-sniffer are presented in Figure 6. The calibration equation can be expressed as follows: ∆intensity (counts) = 2796.9 × (IPA conc. ppb)^0.886^ at R^2^ = 0.992. The dynamic range was confirmed from 0.5 to 1940 ppb. For the IPA gas in ppm levels, the original bio-sniffer (with a dynamic range from 1 to 9060 ppb [25]), could determine a higher concentration than the modified one. In contrast, for the extremely low IPA concentration, the modified bio-sniffer extend the LOD to a sub-ppb level, 0.5 ppb, which was superior to the previous one. Moreover, the average signals that modified the bio-sniffer measured at 0.5 ppb were approximately 1597 counts, still higher than three times the blank standard deviation of 924 counts. Accordingly, the modified bio-sniffer has the potential to detect an IPA lower than 0.5 ppb. We did not explore a lower concentration because 0.5 ppb was the lowest concentration that the standard gas generator could produce. The reproducibility of the modified bio-sniffer was similar to the previous one (Figure S3). For the five cyclic 50 ppb IPA gas measurement, the coefficient of variation value (C.V.) was 0.94%. Table 1 shows the comparison of the detection limit of the IPA gas sensor from the literature. Other IPA sensors such as NiO decorated CeO_2_ [34], SnO_2_ [35,36], carbon nanotubes [37], BiVO_4_ [38], and BiFeO_3_ [39] have a fast response. However, their detection limit was from 1 to 10 ppm, which was much higher than this work.

The modified bio-sniffer sacrifices the upper detection limit from the original 9060 to 1940 ppb because of NADH concentration saturation. The decrease in the upper detection limit is not a problem because, for human breath analysis, the IPA concentration is extremely rare in a ppm-level. This performance could be improved by increasing the concentration of NAD^+^ in the circular buffer and elevating the buffer’s flow rate. In contrast, the modified bio-sniffer extends the LOD to 0.5 ppb, which promotes greater applicability to human exhale analysis and other applications. For example, it may be applied to the analysis of other samples containing IPA in extremely low concentrations, including skin gas [40,41] or VOCs from urine [42].

### 3.4. Breath Sample Measurement

**Figure 7:**
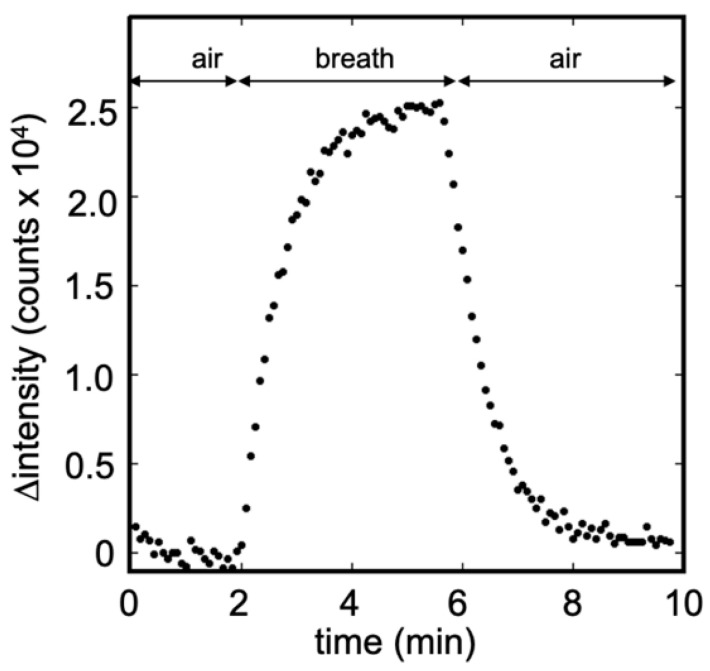
Breath measurement using the modified IPA bio-sniffer. The IPA concentration was detected to be 17.9 ppb in this sample. The pattern of signal temporal change was similar to the standard IPA measurement.

Figure 7 illustrates the temporal change in the fluorescence signal obtained from the breath sample using the modified bio-sniffer. The collected breath was approximately 1 L, which was sufficient for an analysis of 4 min. In this sample, the IPA concentration was determined to be 17.9 ppb, close to our previous investigation of breath IPA level in healthy people, 15.4 ppb. Compared to Figure 6b, the breath sample detection’s fluorescence intensity change pattern was similar to the standard IPA measurement. Consequently, the measurement is not much affected by the presence of the other compounds present in the exhaled air (such as water or CO_2_).

## 4. Conclusions

The IPA bio-sniffer was successfully modified, extending the detection limit to 0.5 ppb, while the LOD is a little lower than 0.5 ppb. The C8855-type PMT exhibits superior photon sensitivity performance for NADH fluorescence detection. It was more important to improve the sensor sensitivity of the multi-core bifurcated optical fiber, which increased the transfer efficiency of excitation light power and enhanced the NADH’s fluorescence. The enlarged fiber probe intensified the collection of the fluorescence. The redesigned flow-cell broadened the space of the enzyme-immobilized membrane in contact with the air sample, contributing to greater NADH accumulation during the reaction. The calibration range is 0.5 ppb up to 1.9 ppm, which covers the broadest range of breath IPA concentration in healthy people and other studied diseases [26,30,43,44,45]. The modified bio-sniffer favorably measures the IPA concentration in a breath sample from the volunteer, confirming its applicability to a real-world exhaled breath analysis. Additionally, the continually circulating buffer keeps the sensor-sensitive surface always wet, which means that the sensing region has 100% humidity. It prevents the measurement from being affected by the moisture of the sample. The expanded detection limit to 0.5 ppb promotes the bio-sniffer as more suitable for human gas analysis, with a greater potential for constructing a clinical non-invasive diagnostic apparatus.

## Figures and Tables

**Table 1:** Comparison of the IPA gas sensors from the literature.

Material	Detection Range (ppm ppb)	Operating Temp. (°C)	Response/Recovery Time (s)	Ref.
SnO_2_ nanorings	1 to 500 ppm	250	6.8/38.6	[35]
SnO_2_ hollow cubes	1 to 1000 ppm	180	1/-	[36]
NiO decorated CeO_2_	1 to 100 ppm	Room temperature	15/19	[34]
Carbon Nanotubes	10 to 1000 ppm	Room temperature	110/152	[37]
BiVO4	1 to 100 ppm	400–500	18/14	[38]
BiFeO3	2 to 100 ppm	240	6/17	[39]
S-ADH/NADH	0.5 to 1940 ppb^3^	Room temperature	180/230	This work

## Supplementary Materials

The following are available online at https://www.mdpi.com/1424-8220/20/23/6827/s1, Figure S1: NADH measurement system. Figure S2: Calibration curves of NADH detection by previous and multi-core type bifurcated fiber probe. Figure S3: Reproducibility of modified bio-sniffer. Table S1: Specifications of C8855 counting unit, H7421-40 counting head and C9692 PMT.
