# Supplementary material for: Ultra-Sensitive Isopropanol Biochemical Gas Sensor (Bio-Sniffer) for Monitoring of Human Volatiles
Source: Sensors (Basel). 2020 Nov 29;20(23):6827. doi: 10.3390/s20236827 (PMC7730524; doi:10.3390/s20236827)
Supplement: Supplementary file 1 [file sensors-20-06827-s001.pdf]

Supplementary Materials

Ultra-Sensitive Isopropanol Biochemical Gas Sensor (Bio-Sniffer) for Monitoring of Human Volatiles

Po-Jen Chien [1], Takuma Suzuki [2], Ming Ye [3], Koji Toma [3], Takahiro Arakawa [3], Yasuhiko Iwasaki [4] and Kohji Mitsubayashi [2,3,*]

[1] Institute of Chemistry, Academia Sinica, No. 128, Sec. 2, Academia Rd., Nankang, Taipei 115, Taiwan; wizgx@gate.sinica.edu.tw
[2] Graduate School of Medical and Dental Sciences, Tokyo Medical and Dental University, 1-5-45 Yushima, Bunkyo-Ku, Tokyo 113-8549, Japan; sensinglabo@gmail.com
[3] Department of Biomedical Devices and Instrumentation, Institute of Biomaterials and Bioengineering, Tokyo Medical and Dental University, 2-3-10 Kanda-Surugadai, Chiyoda-ku, Tokyo 101-0062, Japan; ym@fujiclinic.jp (M.Y.); toma.bdi@tmd.ac.jp (K.T.); arakawa.bdi@tmd.ac.jp (T.A.)
[4] Faculty of Chemistry, Materials and Bioengineering, Kansai University, 3-3-35 Yamate-Cho, Suita-Shi, Osaka 564-0836, Japan; yasu.bmt@kansai-u.ac.jp
* Correspondence: m.bdi@tmd.ac.jp; Tel.: +81-3-5280-8091

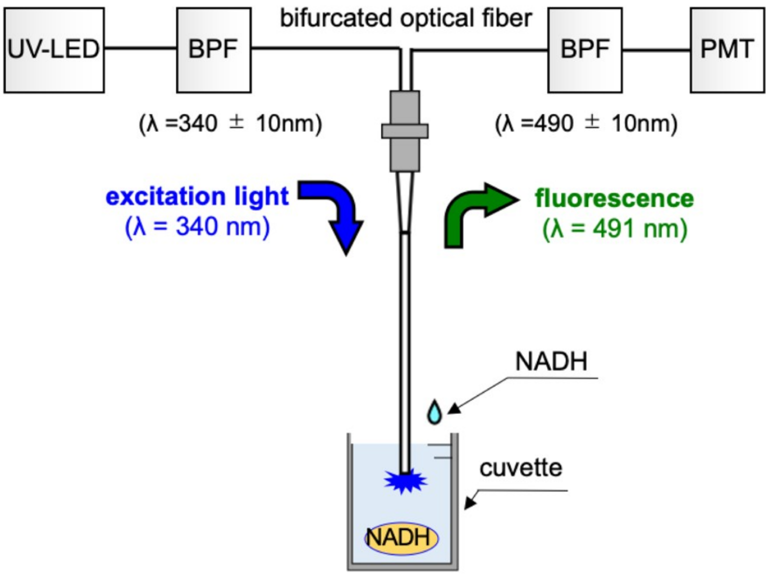

Figure S1. NADH measurement system. The NADH measurement system was composed by an UV-LED, a photomultiplier, an optical fiber probe, and a bifurcated fiber. Two band-pass filters (BPF, λ=340±10 nm and 490±10 nm) were equipped for reducing the interference.

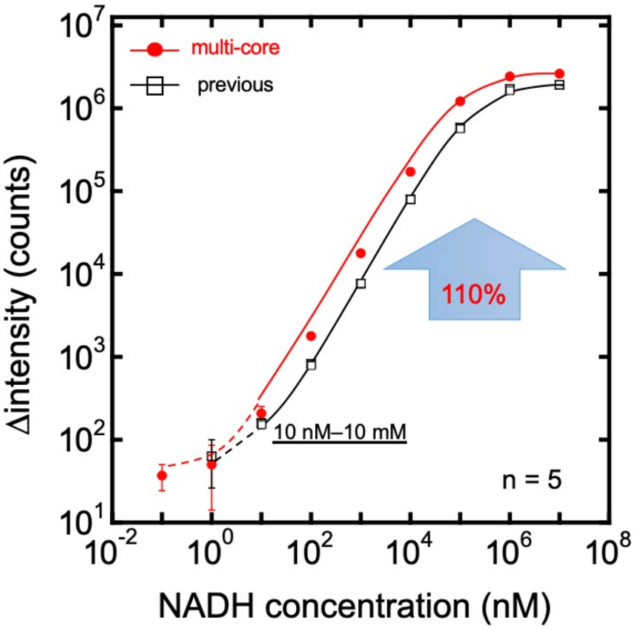

Figure S2. Calibration curves of NADH detection by previous and multi-core type bifurcated fiber probe. The signal intensity measured by the multi-core type showed about more 110% than the previous one. The dynamic range was the same, both were from 10 nM to 10 mM.

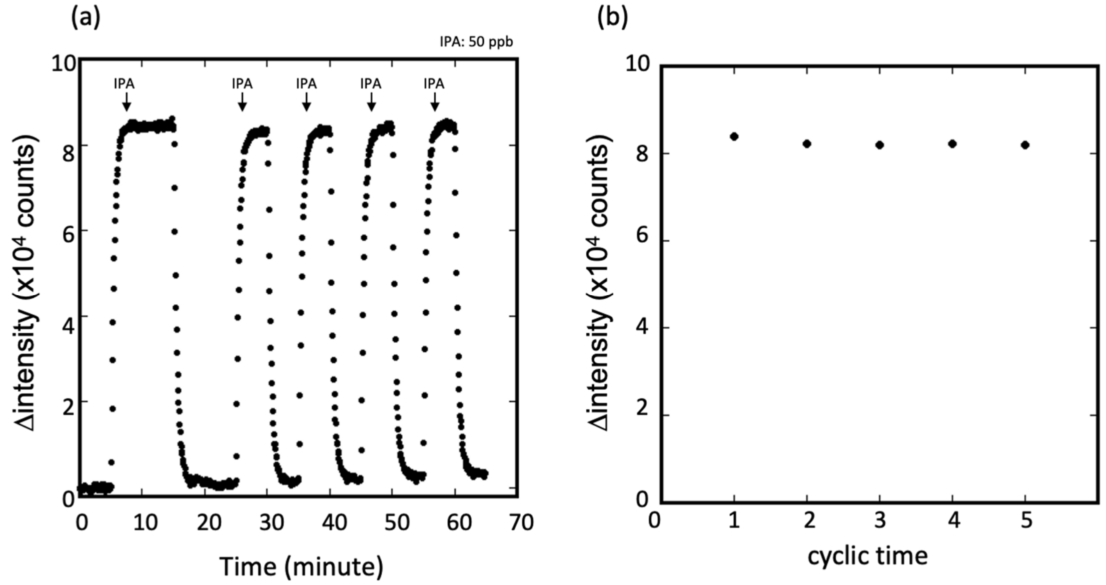

Figure S3. (a) Reproducibility curve of modified bio-sniffer tested by 50 ppb IPA gas. The first cycle measured the IPA gas for 10 minutes, and the second to fourth cycle measured the IPA gas for 5 minutes. (b) The Δintensity of five cyclic measurements. The coefficient of variation was about 0.94%.

Principle of generating different concentrations of IPA gas

The gas generators we employed (PD-1B-2 and custom-made machine) were the calibration gas generation system. The dilution gas is calculated using the following equation (supply from Gastec, Co., Ltd., Japan):

$$F = (K \times Pr \times L) / C \qquad (1)$$

or

$$F = (K \times Dr \times 103) / C \qquad (2)$$

Equation (1) is for the permeation tube, and equation (2) uses a diffusion tube. For generating the different concentrations of IPA gas for calibration, we used diffusion tube with Dr 6.68 at 35 ºC (PD-1B-2, D-01, C: 270 ppb to 9.06 ppm), diffusion tube with Dr 40.4 at 35 ºC (PD-1B-2, D-03, 1.64 ppm to 82.21 ppm), permeation tube with Pr 85.2 at 35 ºC (PD-1B-2, C: 7.7 ppb to 346 ppb, F: 200 ml/min to 8 L/min))and permeation tube with Pr 85.2 at 10 ºC (custom-made machine , C: 0.5 ppb to 10 ppb, F: 200 ml/min to 5 L/min). The K value of IPA is 0.407. By adjusting the different flow of dilution gas (F), we could produce different IPA gas concentrations.

Abbreviation:

F：Flow of dilution gas (mL/min) (PD-1B-2: 200 ml/min to 8 L/min; custom-made machine: 200 ml/min to 5 L/min)

C：Gas concentration for calibration gas (ppm)

Pr：Permeation rate (ng/min/cm)

L：Effective length of tube (cm)

K：Coefficient for converting the gas weight into volume (L/g)

$K = (22.4/M) \times ((273+t)/273) \times (760/P)$, (25ºC 1atm);

M：Molecular weight; t：Temperature

P：Pressure (mmHg)

Table S1 Specifications of C8855 counting unit, H7421-40 counting head and C9692 PMT

**Parameter**	**Description / Value**		
	C8855 counting unit	H7421-40 counting head	C9692
**Signal pulse width**	8 ns or longer	-	100 ns or more
**Max. count rate**	50×10^6	-	3×10^6
**Max. counter capacity**	2^{32} counts / gate	-	2^{32} counts / gate
**Internal counter gate time**	50 μs to 10 s		1 μs to 10 s
**Spectral Response range**	-	300 to 720 nm	185 to 650 nm
**Count Sensitivity**		420 nm: 4.7×10^5 $s^{-1}\cdot pW^{-1}$	-
		550 nm: 7.6×10^5 $s^{-1}\cdot pW^{-1}$	
**Dark Count**	-	100	50
**Photocathode size**	-	-	16 × 18 mm

This information supply from Hamamatsu Corporation, Japan
